# Alternative lengthening of telomeres is seen in a proportion of oligodendrogliomas and is associated with a worse prognosis

**DOI:** 10.1093/noajnl/vdae006

**Published:** 2024-02-09

**Authors:** Zhi-Feng Shi, Kay Ka-Wai Li, Danny Tat-Ming Chan, Ying Mao, Ho-Keung Ng

**Affiliations:** Department of Neurosurgery, Huashan Hospital, Fudan University, Shanghai, China; Hong Kong and Shanghai Brain Consortium (HSBC), Hong Kong, China; Department of Anatomical and Cellular Pathology, The Chinese University of Hong Kong, Shatin, Hong Kong, China; Division of Neurosurgery, Department of Surgery, The Chinese University of Hong Kong, Shatin, Hong Kong, China; Department of Neurosurgery, Huashan Hospital, Fudan University, Shanghai, China; Hong Kong and Shanghai Brain Consortium (HSBC), Hong Kong, China; Hong Kong and Shanghai Brain Consortium (HSBC), Hong Kong, China; Department of Anatomical and Cellular Pathology, The Chinese University of Hong Kong, Shatin, Hong Kong, China

**Oligodendrogliomas are known to be mutated for telomerase reverse transcriptase promoter (TERTp), and in this report, we evaluated 112 IDH-mutant, 1p19q codeleted oligodendrogliomas for alternative lengthening of telomeres (ALT) by fluorescence in situ hybridization (FISH), and FISH for copy-number changes of CDKN2A, MYC, PDGFRA, EGFR, chromosomes+7/10, and TERT rearrangement. Enigmatically, 35.7% of cases were ALT positive in spite of the vast majority of them being TERTp mutant. ALT was associated with a shorter progression-free survival (*P* = .009) and remained an independent prognosticator in multivariate analysis. ALT was also associated with MYC amplification. ALT-positive cases were further examined with targeted sequencing. No genes were found to be of prognostic significance in this group.**

Maintenance of telomere length is an important mechanism of gliomagenesis and is generally believed to be achieved by 2 different mechanisms: activation of telomerase reverse transcriptase (TERT) by promoter mutations or a telomerase-independent mechanism known as alternative lengthening of telomeres (ALT) which relies on the homologous recombination of telomeric regions and results in a heterogeneous length and sequence composition. Oligodendrogliomas are well known to be mutated for TERT promoter (TERTp), which is enigmatically a diagnostic criterion for molecular glioblastoma by the World Health Organization (WHO) 2021 classification. It is believed that gliomas that are TERTp negative maintain their telomere lengths by means of ALT, which is usually reflected by mutations of alpha-thalassemia/mental retardation, X-linked (ATRX). However, oligodendrogliomas are known to be non-mutated for ATRX unlike astrocytomas.^1^ TERT rearrangement has also been shown to be an alternative way of TERT activation.^2^ We revisited telomere maintenance mechanisms in oligodendrogliomas. We evaluated 112 oligodendrogliomas of both Grades 2 (*n* = 87) and 3 (*n* = 25) from our hospitals. All cases were isocitrate dehydrogenase (IDH) mutant, 1p19q codeleted, and 93% were TERTp mutated by Sanger sequencing (0% non-mutated, 7% sequencing unsuccessful). We studied them using formalin-fixed, paraffin-embedded (FFPE) by fluorescence in situ hybridization (FISH) for ALT and TERT rearrangements as previously performed by others and us.^2–5^ FISH was also performed for CDKN2A/B homozygous deletion (HD), EGFR, MYC, and PDGFRA amplifications, and chromosomes +7/–10 as previously done by us.^6^ Detailed methods were available in Supplementary Materials. Briefly, for FISH for ALT, we used the Telomere PNA FISH kit (K532511, Dako). Cases were considered ALT positive when (1) they displayed ultra-bright nuclear foci (telomere FISH signal of 10-fold greater than the signals of individual non-neoplastic cells), and (2) ≥5% of tumor cells exhibited large, very bright intranuclear foci of telomere FISH signals.^2–5^ Areas of necrosis were excluded from analysis. For controls for ALT and other FISH biomarkers, we used positive cases from previous studies^3,6,7^ and normal brain tissues as negative controls. Forty cases (35.7%) showed ALT (Figure 1A and Supplementary Figures 1 and 2) and for cases with available information, ALT positivity was associated with worse progression-free survival (PFS; *P* = .009; Figure 1B) and remained an independent prognosticator in multivariate analysis (Supplementary Table 1). Amplifications of MYC (6.3%) and PDGFRA (10.7%) were detected and 11.6% showed CDKN2A/B HD and none showed +7 or –10, and all these had no prognostic significance (Supplementary Figure 1). TERT rearrangement was only seen in 10.7% and had no prognostic significance. ALT was found to be associated with MYC amplification (*P* = .004) and not with histological grades (*P* = .361) or other biomarkers (Supplementary Table 2).

**Figure 1.**
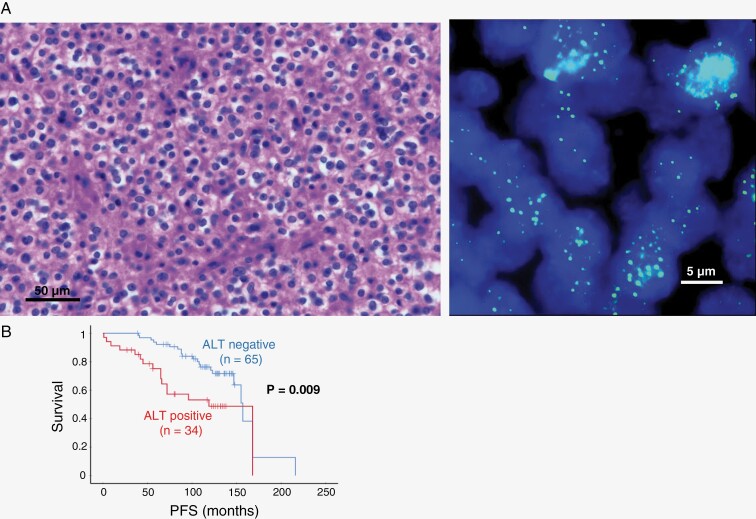
(A) (Left) Grade 2, IDH-mutant, 1p19q-codeleted oligodendroglioma in the frontal lobe (M/41y) (×400). (Right) Large, ultra-bright telomeric FISH signals indicate ALT phenotype. (B) ALT is associated with a shorter PFS in this cohort.

As there have been many studies on genomic sequencing of oligodendrogliomas already, we only further studied the ALT-positive cases by targeted DNA sequencing (Supplementary Table 3), including genes described to be associated with poorer prognosis in oligodendrogliomas, mostly in Grade 3 cases, namely CIC, FUBP1, TCF12, PIK3CA, and NOTCH1 (Supplementary Tables 4 and 5).^1^ ATRX mutation as expected was found only in 1 case and no p53 mutation was found. Also, mutations of H3.3 and H3.1 genes, which were not identified as chromatin remodeling genes, are known to be able to trigger ALT.^8^ Mutations of SMARCAL1, another gene that has been described to contribute ALT telomere maintenance,^2^ was also not found. No gene mutations were of prognostic significance in this group.

WHO classification 2021 has not yet designated definite molecular criteria for anaplastic oligodendroglioma.^1^ Deletions in CDKN2A/B, increased MYC signaling, and mutations in FUBP1, NOTCH1, PIK3CA, and TCF12 have all been associated with tumor progression or poorer survival in oligodendroglioma.^1^ CDKN2A/B HD is now established by WHO as a molecular criterion for IDH-mutant Grade 4 astrocytomas. For IDH-mutant, 1p19q-codeleted oligodendrogliomas, CDKN2A/B HD was not seen in Grade 2 tumors and was associated with poorer prognosis only in Grade 3 tumors according to a major study.^9^ Our results show that ALT is enigmatically seen in a proportion of both Grades 2 and 3 oligodendrogliomas in spite of the presence of TERTp mutations, and is associated with a worse prognosis (Supplementary Table 6). The limitation of our study was that we were unable to test our results for ALT with an alternative method. The other method for testing for ALT is the C-circle test. However, this method requires fresh tissues,^2^ of which we could not perform with the FFPE tissues that we only had for these cases. In our study, ALT is also associated with MYC amplification, and MYC was a major mechanism for more aggressive behavior in oligodendroglioma in a major study.^10^ Our results show mechanisms for telomere maintenance in gliomas are complex. ALT can be studied by FISH in routine laboratories and will be a useful adjunct for prognostication in oligodendrogliomas and a supplement for other histological or molecular parameters.

## Supplementary Material

vdae006_suppl_Supplementary_Figures_1-2_Tables_1-6
