# Supplementary material for: Alternative lengthening of telomeres is seen in a proportion of oligodendrogliomas and is associated with a worse prognosis
Source: Neurooncol Adv. 2024 Feb 9;6(1):vdae006. doi: 10.1093/noajnl/vdae006 (PMC10873776; doi:10.1093/noajnl/vdae006)
Supplement: vdae006_suppl_Supplementary_Figures_1-2_Tables_1-6 [file vdae006_suppl_supplementary_figures_1-2_tables_1-6.pdf]

Supplementary Figure and Table Legends

Supplementary Figure 1. Oncoprint showing the clinical and molecular characteristics of 112 IDH-mutant, 1p19q-codeleted oligodendrogliomas.

Supplementary Figure 2. A. 38/F, frontal lobe IDH mutant, 1p19q co-deleted Grade 3 oligodendroglioma, with FISH for ALT. Arrows show microvascular proliferation. B. 61/M, frontal lobe IDH mutant 1p19q co-deleted oligodendroglioma, Grade 2, with FISH for ALT. C. 27/M, frontal lobe IDH mutant 1p19q co-deleted Grade 3 oligodendroglioma, with FISH for ALT. D. 41/M, frontal lobe IDH mutant 1p19q co-deleted Grade 2 oligodendroglioma, with FISH for ALT. E. 39/M, periventricular IDH mutant 1p19q co-deleted Grade 2 oligodendroglioma, with FISH for ALT.

Supplementary Table 1. Multivariate analysis of clinical and molecular features of IDH-mutant, 1p19q-codeleted gliomas.

Supplementary Table 2. Correlation between ALT and molecular features in IDH-mutant, 1p19q-codeleted gliomas.

Supplementary Table 3. List of genes studied by target sequencing.

Supplementary Table 4. Frequency of mutations in 40 ALT-positive, IDH-mutant, 1p19q-codeleted gliomas.

Supplementary Table 5. Gene mutations and survival in 40 ALT-positive, IDH-mutant oligodendrogliomas.

Supplementary Table 6. Clinical characteristics of 112 patients of IDH-mutant, 1p19q-codeleted gliomas.

Online Materials and Methods

Supplementary Figure 1

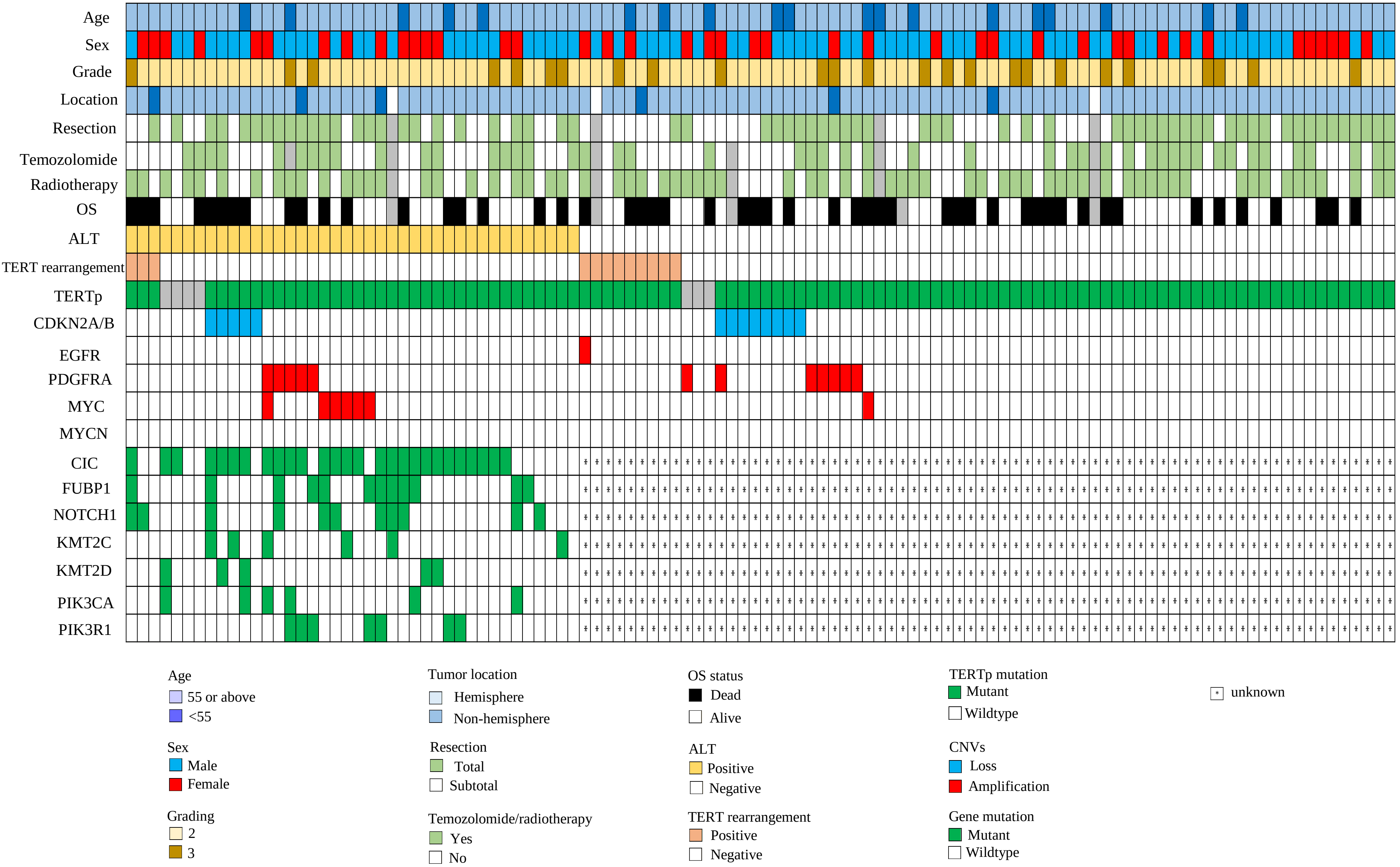

See also online Supplementary Tables 4 and 5.

Supplementary Figure 2A

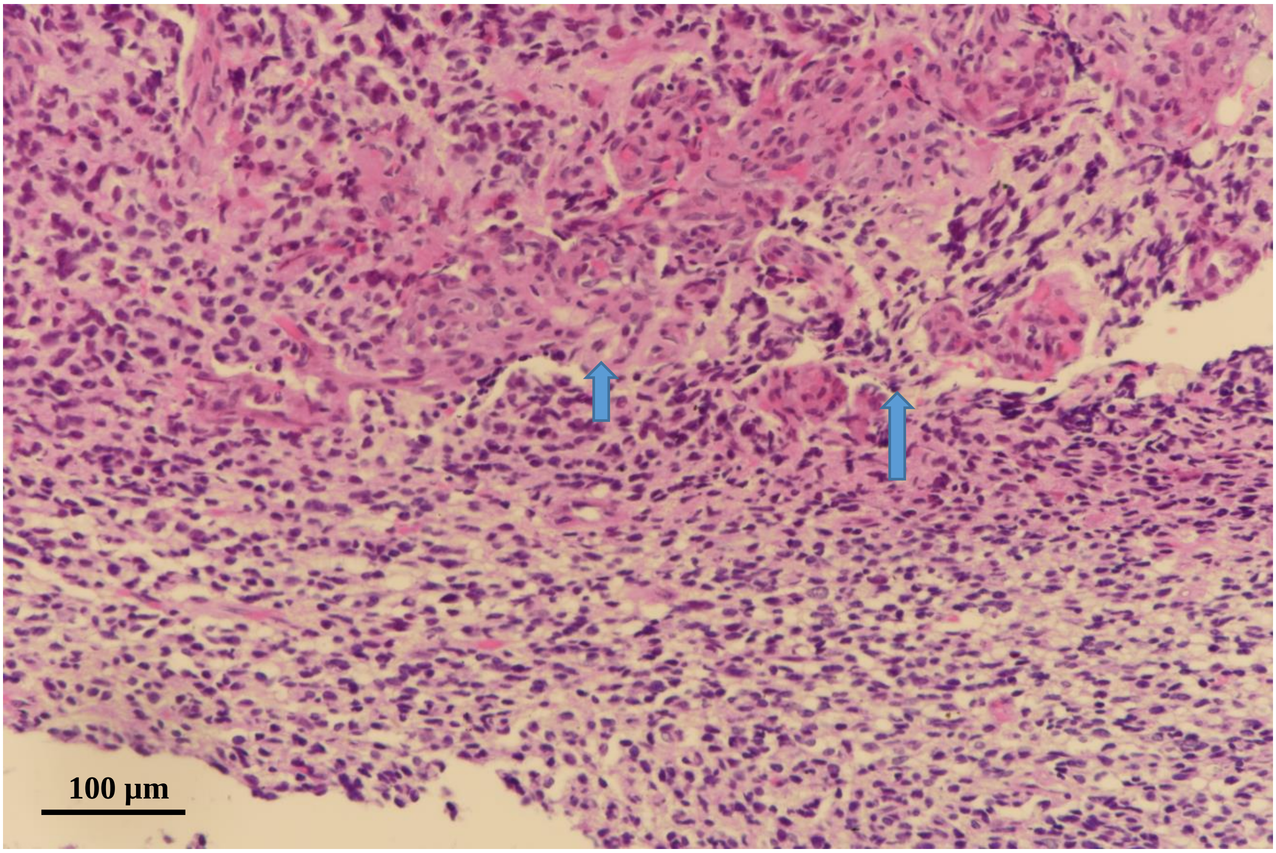

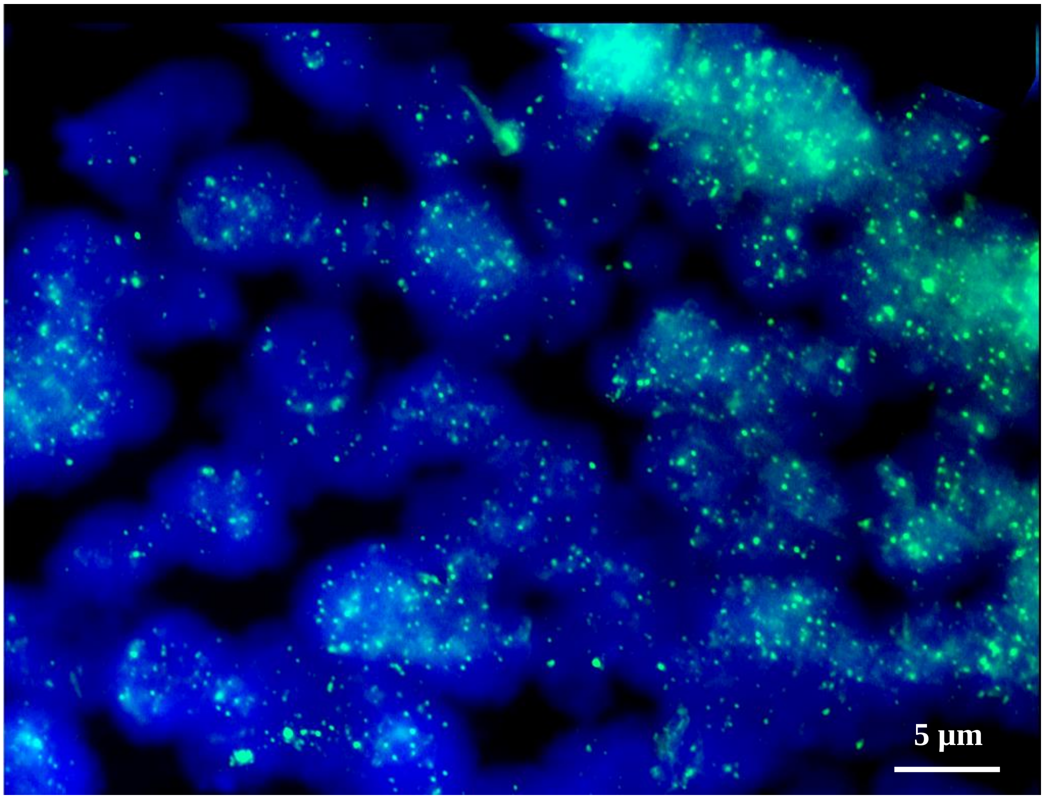

38/F, frontal lobe IDH mutant, 1p19q co-deleted Grade 3 oligodendroglioma, with FISH for ALT. Arrows show microvascular proliferation.

Supplementary Figure 2B

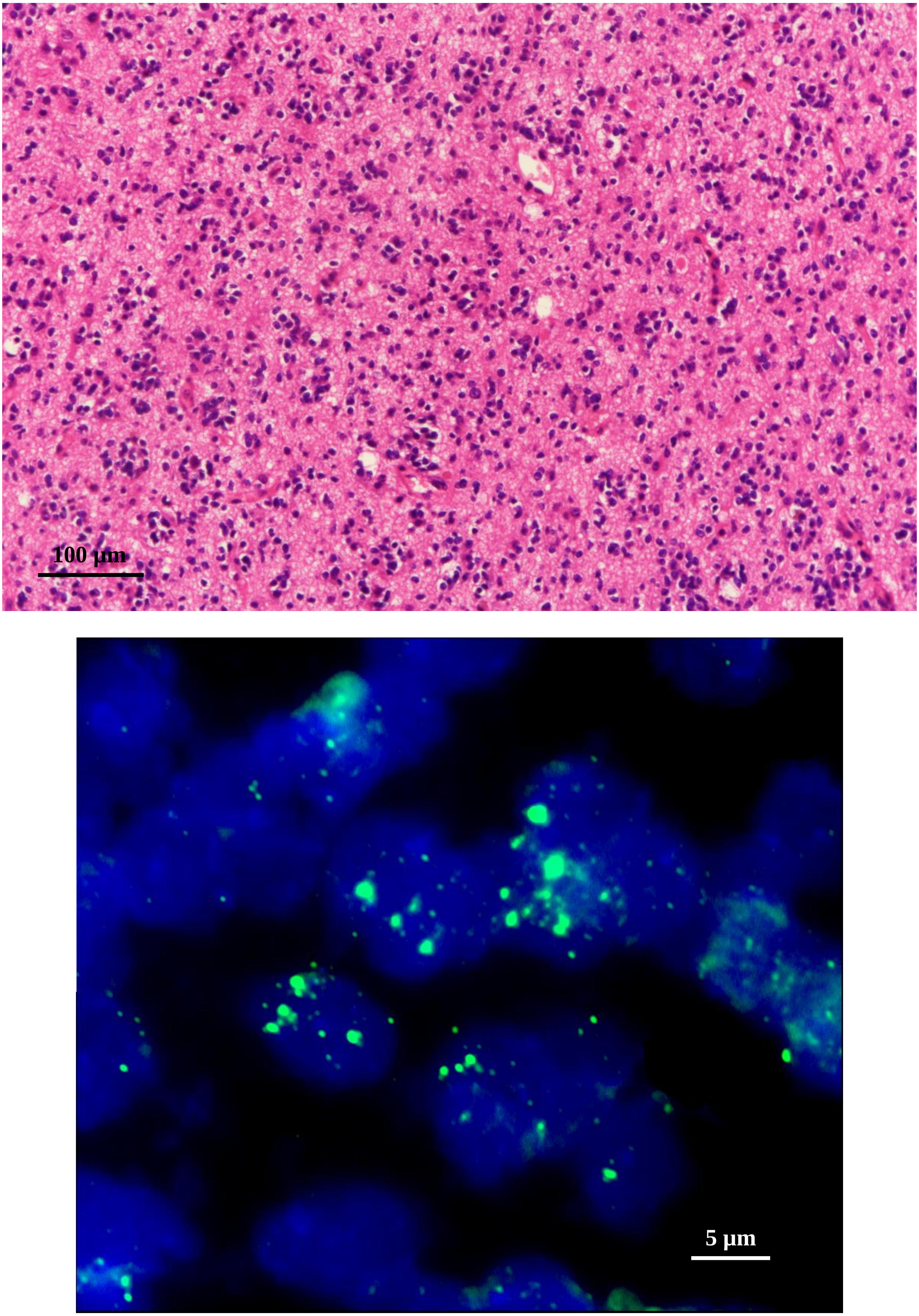

61/M, frontal lobe IDH mutant 1p19q co-deleted oligodendroglioma, Grade 2, with FISH for ALT.

Supplementary Figure 2C

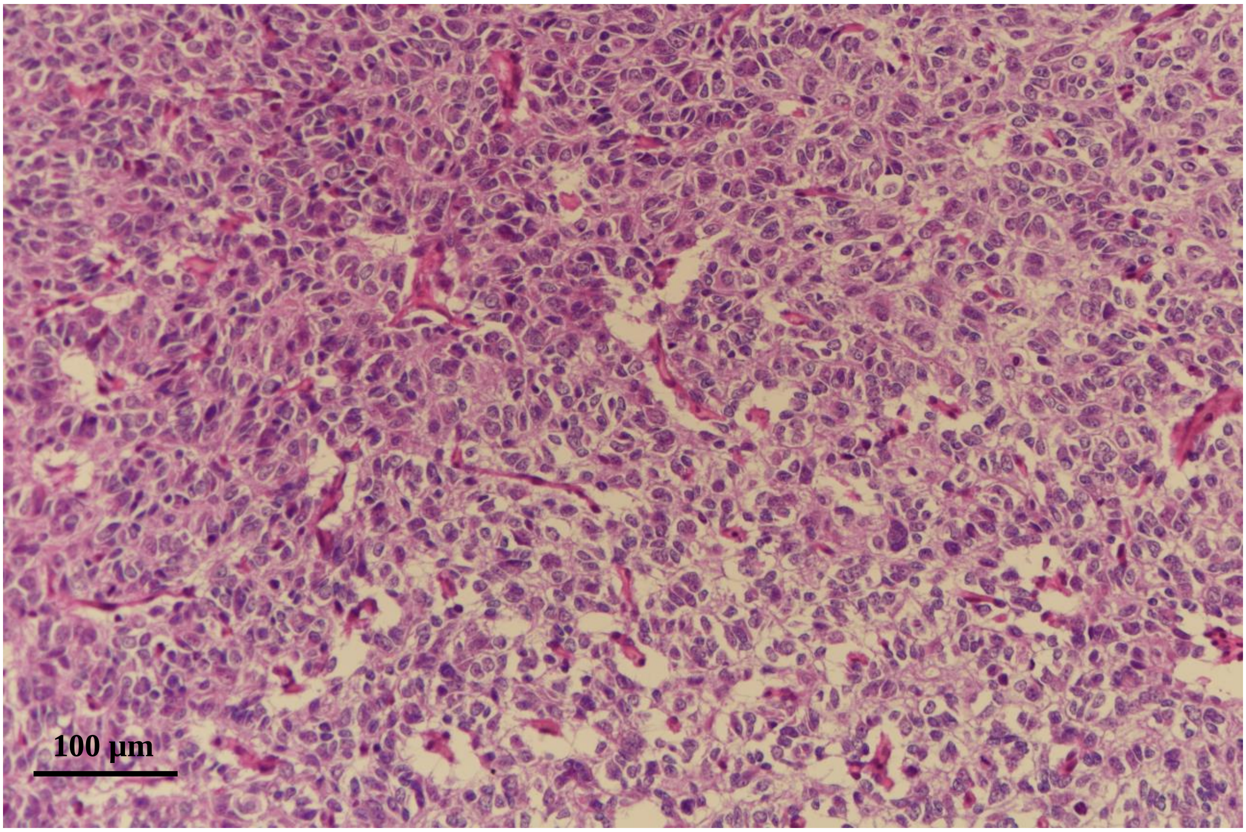

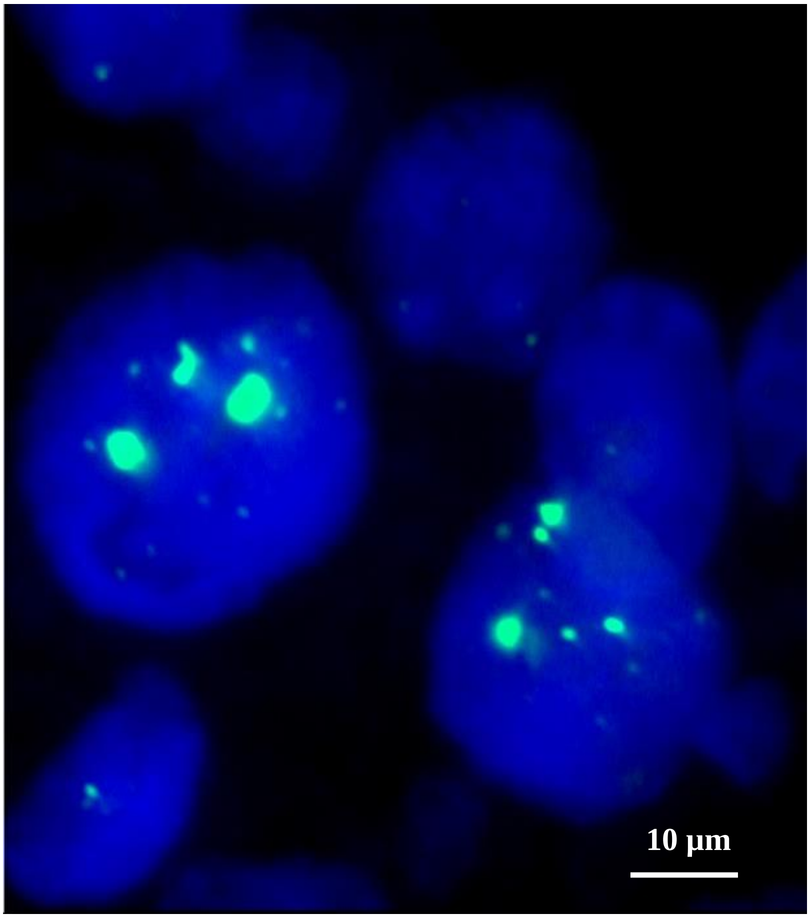

27/M, frontal lobe IDH mutant 1p19q co-deleted Grade 3 oligodendroglioma, with FISH for ALT.

Supplementary Figure 2D

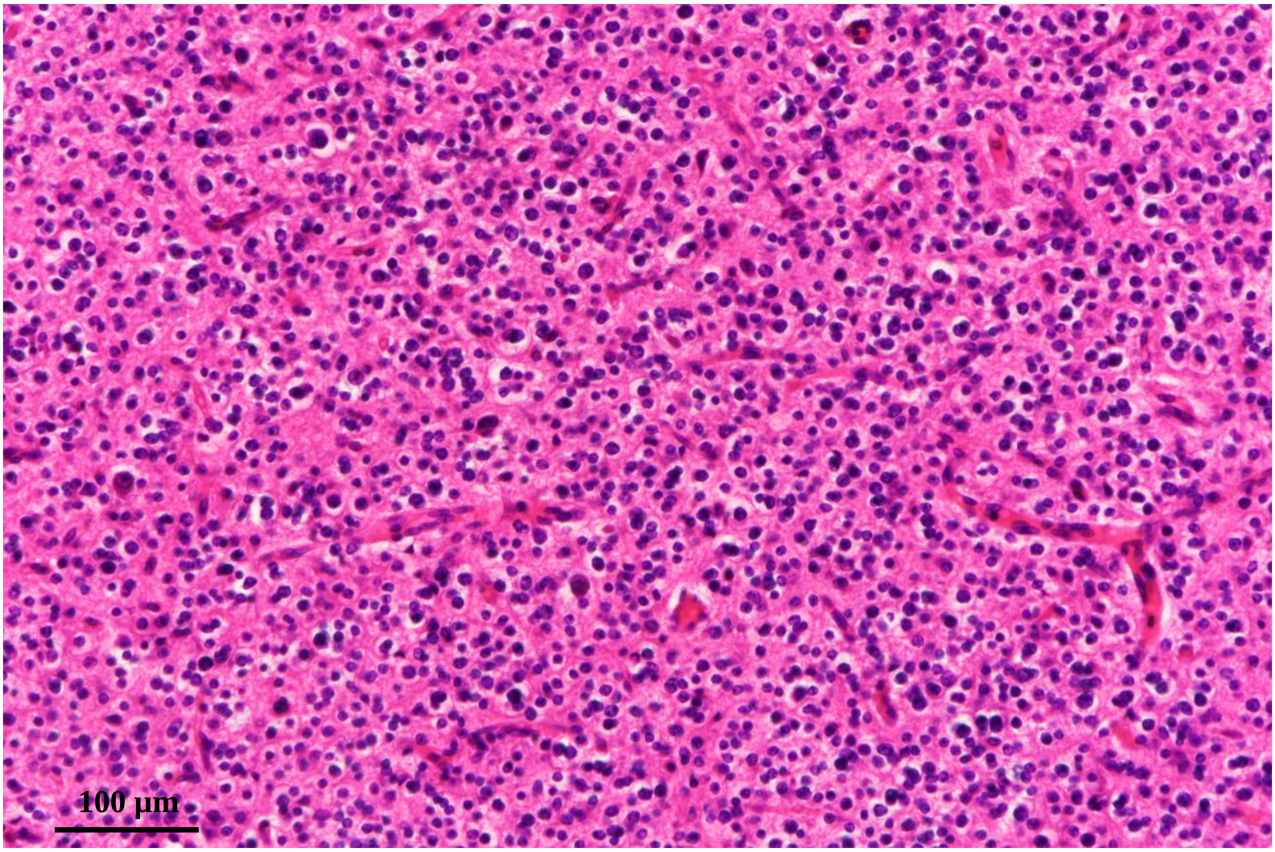

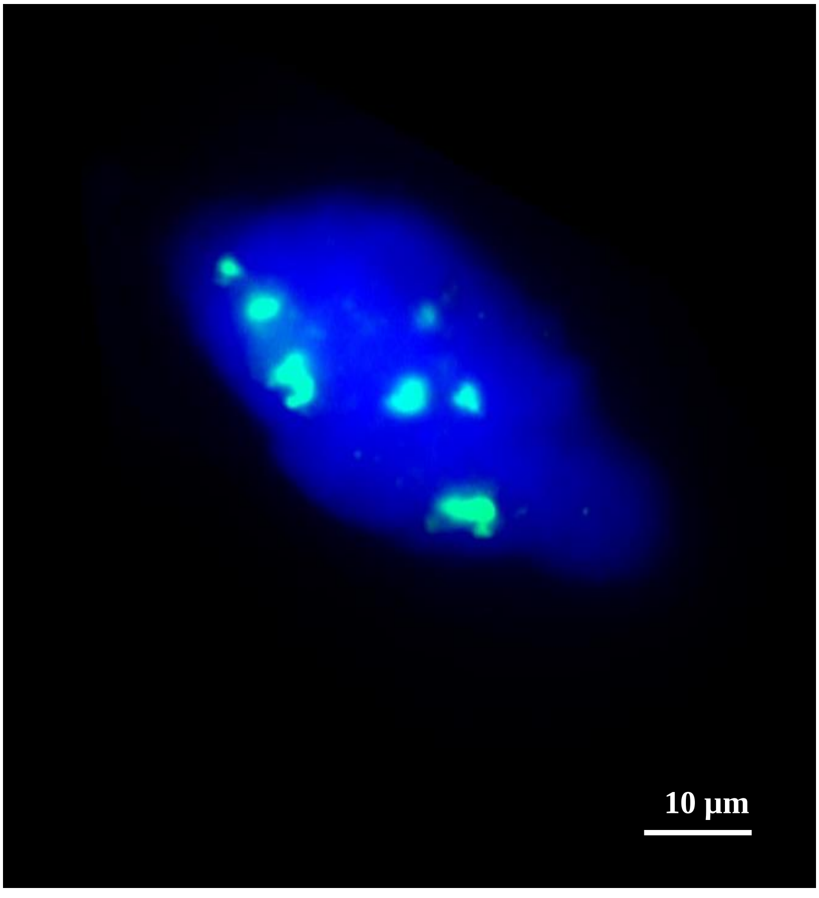

41/M, frontal lobe IDH mutant 1p19q co-deleted Grade 2 oligodendroglioma, with FISH for ALT.

Supplementary Figure 2E

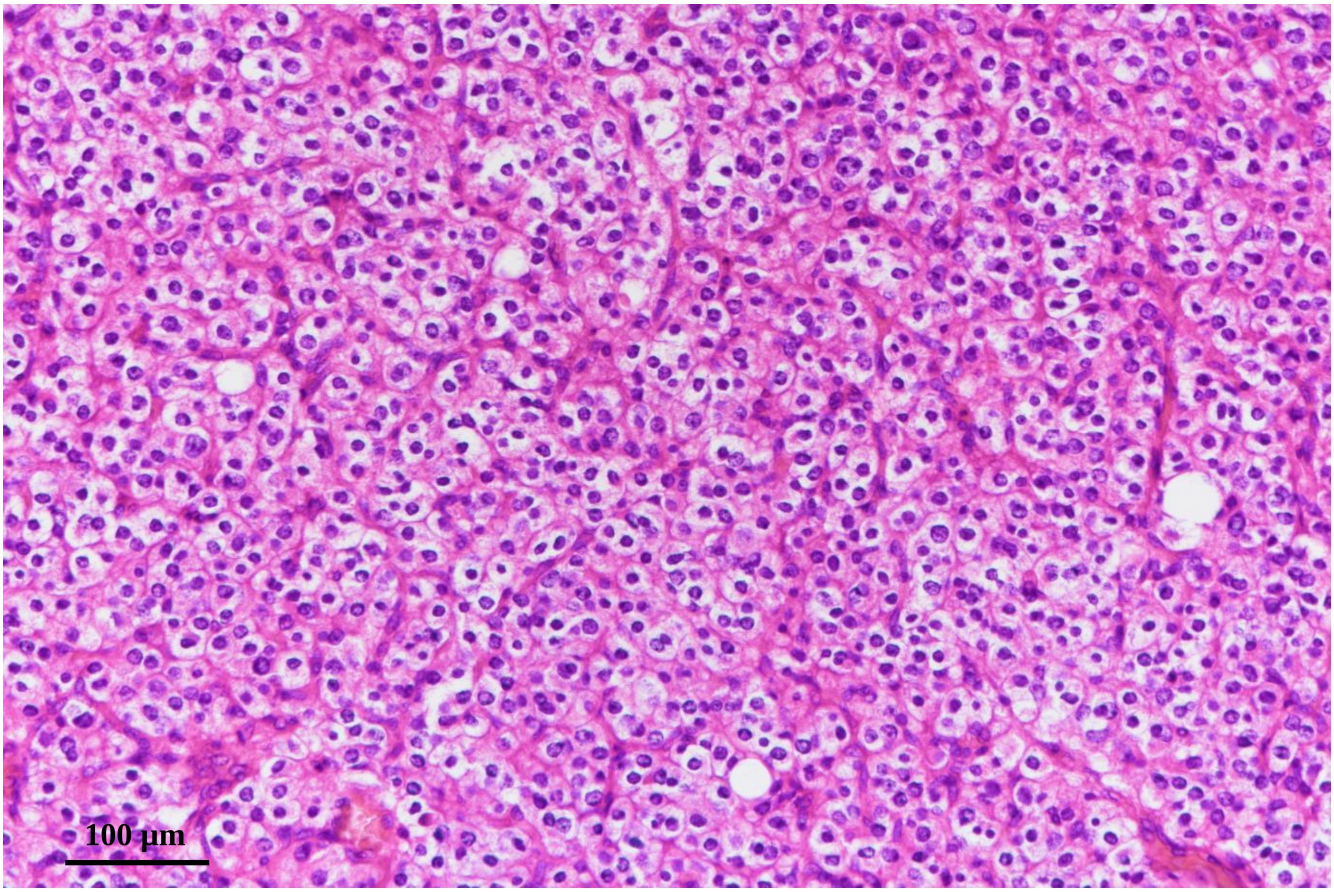

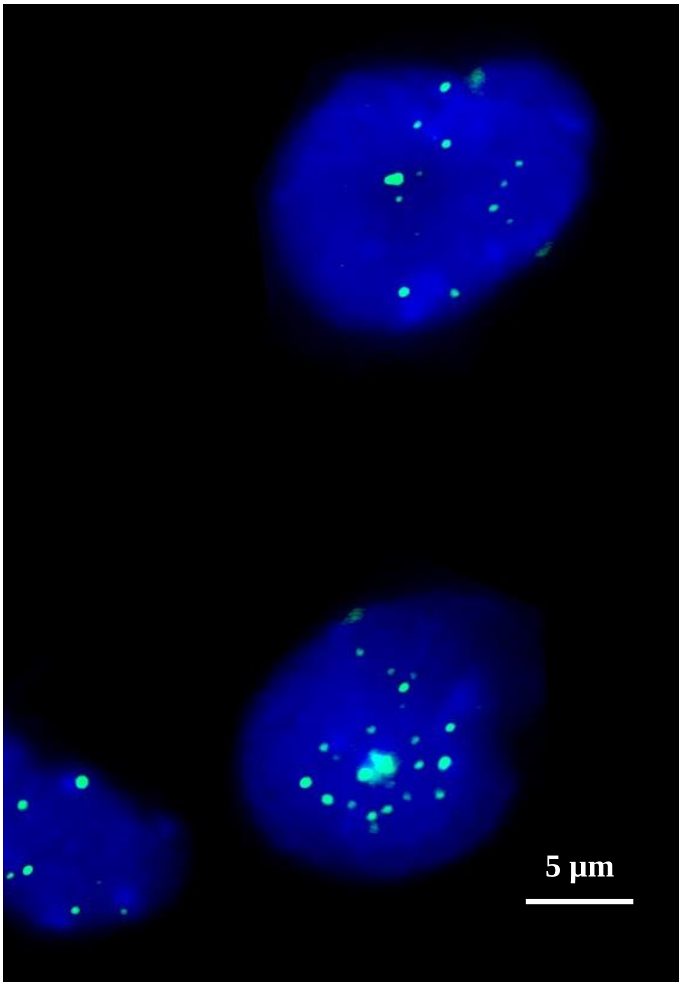

39/M, periventricular IDH mutant 1p19q co-deleted Grade 2 oligodendroglioma, with FISH for ALT.

Supplementary Table 1. Multivariate analysis of clinical and molecular features of IDH-mutant, 1p19q-codeleted gliomas.			
Features		PFS	
		HR (95% CI)	p values
Age			
	<55 years old	1	0.991
	≥55 years old	0.995 (0.392-2.521)	
Sex			
	Male	1	0.639
	Female	0.841 (0.408-1.734)	
Grading			
	2	1	0.384
	3	0.684 (0.291-1.608)	
Location			
	Hemisphere	1	0.703
	Non-hemisphere	0.671 (0.086-5.222)	
Operation			
	Total resection	1	**0.003**
	Non-total resection	3.343 (1.529-7.312)	
Temozolomide			
	Yes	1	0.466
	No	0.756 (0.357-1.603)	
Radiotherapy			
	Yes	1	0.639
	No	0.828 (0.377-1.818)	
ALT			
	Negative	1	**0.002**
	Positive	3.462 (1.598-7.498)	

Supplementary Table 2. Correlation between ALT and molecular features in IDH-mutant, 1p19q-codeleted gliomas.

Features		Number of cases	ALT negative (n, %)	ALT positive (n, %)	p values
TERT rearrangement					
	Yes	12 (10.7%)	9 (8.0%)	3 (2.7%)	0.412
	No	100 (89.3%)	63 (56.3%)	37 (33.0%)	
CDKN2A/B					
	Homozygous deletion (HD)	13 (11.6%)	8 (7.1%)	5 (4.5%)	0.826
	No HD	99 (88.4%)	64 (57.1%)	35 (31.3%)	
EGFR					
	Amplification	1 (0.9%)	1 (0.9%)	0 (0%)	0.454
	No amplification	111 (99.1%)	71 (63.4%)	40 (35.7%)	
MYC					
	Amplification	7 (6.3%)	1 (0.9%)	6 (5.4%)	**0.004**
	No amplification	105 (93.8%)	71 (63.4%)	34 (30.4%)	
PDGFRA					
	Amplification	12 (10.7%)	7 (6.3%)	5 (4.5%)	0.649
	No amplification	100 (89.3%)	65 (58.0%)	35 (31.3%)	

Supplementary Table 3. List of genes studied by target sequencing.

ABCB1	ABCC9	ADAM29	AKT1	ATRX	BCOR
BCORL1	BRAF	CCND1	CCND2	CCND3	CDH18
CDK4	CDK6	CDKN2A	CDKN2B	CDKN2C	CIC
COL1A2	CSF1R	CTNNB1	DDX3X	DRD5	EGFR
ERBB2	ERBB3	ERBB4	FAT1	FGFR1	FGFR2
FGFR3	FGFR4	FUBP1	GABRA6	H3F3A	HDAC9
HIST1H3B	HIST1H3C	HMCN1	HRAS	IDH1	IDH2
KDR	KEL	KIT	KLF4	KMT2B	KMT2C
KMT2D	KRAS	LZTR1	MDM2	MDM4	MET
MLH1	MSH2	MSH6	MTOR	MYC	MYCN
NF1	NF2	NLRP5	NOTCH1	NRAS	PBRM1
PDGFRA	PDGFRB	PIK3CA	PIK3CG	PIK3R1	PIK3R2
PMS2	POLE	PPM1D	PTCH1	PTEN	PTPN11
PTPRD	RB1	ROS1	SCN9A	SEMA3C	SEMG1
SETD2	SMARCAL1	SMO	SPTA1	STAG2	TCF12
TCHH	TP53				

Supplementary Table 4. Frequency of mutations in 40 ALT-positive, IDH-mutant, 1p19q-codeleted gliomas.

Genes	Total cases (n, %)	Grade		
		2 (n, %)	3 (n,%)	p value
ATRX	1 (2.5%)	1 (2.5%)	0 (0%)	0.641
CIC	27 (67.5%)	24 (60.0%)	3 (7.5%)	0.125
FUBP1	12 (30.0%)	9 (22.5%)	3 (7.5%)	0.414
NOTCH1	11 (27.5%)	9 (22.5%)	2 (5.0%)	0.944
PIK3CA	6 (15.0%)	4 (10.0%)	2 (5.0%)	0.268
PIK3R1	7 (17.5%)	5 (12.5%)	2 (5.0%)	0.396
ROS1	5 (12.5%)	4 (10.0%)	1 (2.5%)	0.875
TCF12	2 (5.0%)	2 (5.0%)	0 (0%)	0.504
TP53	0 (0%)	0 (0%)	0 (0%)	NA

Supplementary Table 5. Gene mutations and survival in 40 ALT-positive, IDH-mutant oligodendrogliomas.

Genes	Total (n)	p value	
		PFS	OS
CIC	27 (67.5%)	0.308	0.364
FUBP1	12 (30.0%)	0.377	0.497
NOTCH1	11 (27.5%)	0.704	0.465
PIK3CA	6 (15.0%)	0.384	0.871
PIK3R1	7 (17.5%)	0.141	0.299

* Only 2 TCF12 mutations were found.

Supplementary Table 6. Clinical characteristics of 112 patients of IDH-mutant, 1p19q-codeleted gliomas.

Features		Number of cases (n=112)	Frequency (%)	PFS (p value)	OS (p value)
Age					
	<55 years old	93	83.0	0.477	**0.032**
	≥55 years old	19	17.0		
Sex					
	Male	71	63.4	0.544	0.985
	Female	41	36.6		
Location					
	Hemisphere	103	92.0	0.728	**0.009**
	Non-hemispheric	6	5.4		
	Not available	3	2.7		
Operation					
	Gross total resection	66	58.9	**0.030**	0.090
	Non-total resection	42	37.5		
	Not available	4	3.6		
Temozolomide					
	Yes	47	42.0	0.946	**0.020**
	No	59	52.7		
	Not available	6	5.4		
Radiotherapy					
	Yes	68	60.7	0.732	**0.013**
	No	39	34.8		
	Not available	5	4.5		
ALT					
	Yes	40	35.7	**0.009**	0.728
	No	72	64.3		
TERT rearrangement					
	Yes	12	10.7	0.552	0.168
	No	100	89.3		
CDKN2A/B					
	Deletion	13	11.6	0.829	0.304
	No deletion	99	88.4		
EGFR					
	Amplification	1	0.9	0.098	0.113
	No amplification	111	99.1		
MYC					
	Amplification	7	6.3	0.207	0.858
	No amplification	105	93.8		
PDGFRA					
	Amplification	12	10.7	0.419	0.912
	No amplification	100	89.3		

Supplementary Online Materials and Methods

FISH analyses

FISH studies were performed for ALT, EGFR, MYC, PDGFRA and TERT-rearrangement as previously used by us and others. ALT phenotype was examined with the Telomere PNA FISH kit (K532511, Dako) [1, 2]. TERT-rearrangement was assessed with FISH break-apart probes we reported in previous publications and the probes were directly labeled [1, 2]. Other FISH methods were also used in our previous studies [1-5]. Vysis LSI CDKN2A SpectrumOrange/CEP 9 SpectrumGreen Probes (Vysis) was used to investigate CDKN2A deletion. EGFR amplification was detected with the BAC clone (CTD-2199A14) which consists of the genomic sequences of 7p11.2 and a centromere probe (CEP7, Vysis). MYC amplification was detected with the BAC clone CTD-3056O22 which spans the genomic region 8q24.21 and a centromere probe (CEP8, Vysis). For PDGFRA amplification, PDGFRA probes (CTD-2054G11 and RP11-231C18) and centromere probe (CEP 4, Vysis) were used. In brief, tumors areas on 4-μm thickness FFPE sections were identified and marked for evaluation. Tissue sections were then deparaffinized, treated with sodium thiocyanate, digested with pepsin, rinsed and dehydrated. The labeled probes were denatured and hybridized to the sections overnight. Sections were then washed, stained with Vectashield mounting medium and visualized under a Zeiss Axioplan fluorescence microscope. At least 100 non-overlapping signals were counted and analysed in each case. Tumors were considered ALT-positive when (1) they displayed ultrabright nuclear foci (telomere FISH signal of 10-fold greater than the signal of individual non-neoplastic cells); and (2) $\geq$ 5% of tumour cells exhibited large, very bright intranuclear foci of telomere FISH signals [6-8]. Areas of necrosis were excluded from analysis. TERT-rearrangement was considered when break-apart signal in samples was found in >5% of evaluated nuclei [1-2, 8]. EGFR, MYC and PDGFRA amplifications were recorded when >5% examined cells displayed many tight clusters or a ratio of target to reference signal >2 [3, 9]. CDKN2A homozygous deletion was recorded when >20% of tumor cells showed loss of two signals [10]. For positive controls for the FISH biomarkers, we used FFPE sections from cases known to be aberrated for the individual FISH markers from previous publications from our group [1, 2, 11]. For negative controls, we used sections from normal brain.

Targeted sequencing

For targeted sequencing, a panel of customized genes commonly altered in CNS tumors was used [1, 2, 4]. DNA was extracted with GeneRead DNA FFPE kit (Qiagen). DNA were then evaluated with QIAseq DNA QuantiMIZE Assay (Qiagen) to check for quality and quantity. Library preparation was completed with a custom QIAseq Targeted DNA Panel, that examined coding exons or hotspots of CNS relevant genes (Supplementary Table 3). The DNA libraries were then qualified prior to DNA sequencing with MiSeq v3 (Illumina).

Paired-end reads were aligned to the hg19 (GRCh37) build of the human reference genome with BWA-MEM algorithm on GeneGlobe platform (Qiagen). smCounter2 and wANNOVAR were used in variant calling and annotation respectively. Variants that met the following criteria were excluded for further analysis: 1. not passing quality filters; 2. variant allele fractions $\leq$10%; 3. variant allele counts $\leq$5, or 4. minor allele frequencies >1% in overall human population or East Asians or documented in public databases (1000 Genomes, ExAc, gnomAD exome and genome databases).

Online references

1. Liu EM, Shi ZF, Li KK, Malta TM, Chung NY, Chen H, Chan JY, Poon MF, Kwan JS, Chan DT, Noushmehr H, Mao Y, Ng HK. Molecular landscape of IDH-wild type, pTERT-wild type adult glioblastomas. Brain Pathol. 2022; 32(6): e13107.
2. Ng HK, Li KK, Chung NY, Chan JY, Poon MF, Wong QH, Kwan JS, Poon WS, Chen H, Chan DT, Shi ZF, Mao Y. Molecular landscapes of longitudinal NF2/22q and non-NF2/22q meningiomas show different life histories. Brain Pathol. 2023; 33(3): e13120.
3. Yang RR, Li KK, Liu APY, Chen H, Chung NY, Chan AKY, Li F, Chan DT, Mao Y, Shi ZF, Ng HK. Low-grade BRAF V600E mutant oligodendroglioma-like tumors of children may show EGFR and MET amplification. Brain Pathol. 2021; 31(1): 211-214.
4. Wong QH, Li KK, Wang WW, Malta TM, Noushmehr H, Grabovska Y, Jones C, Chan AK, Kwan JS, Huang QJ, Wong GC, Li WC, Liu XZ, Chen H, Chan DT, Mao Y, Zhang ZY, Shi ZF, Ng HK. Molecular landscape of IDH-mutant primary astrocytoma Grade IV/glioblastomas. Mod Pathol. 2021; 34(7): 1245-1260.
5. Chan AK, Shi ZF, Li KK, Wang WW, Chen H, Chung NY, Chan DT, Poon WS, Loong HH, Liu XZ, Zhang ZY, Mao Y, Ng HK. Combinations of Single-Gene Biomarkers Can Precisely Stratify 1,028 Adult Gliomas for Prognostication. Front Oncol. 2022; 12: 839302.
6. Heaphy CM, Subhawong AP, Hong SM, Goggins MG, Montgomery EA, Gabrielson E, Netto GJ, Epstein JI, Lotan TL, Westra WH, Shih IeM, Iacobuzio-Donahue CA, Maitra A, Li QK, Eberhart CG, Taube JM, Rakheja D, Kurman RJ, Wu TC, Roden RB, Argani P, De Marzo AM, Terracciano L, Torbenson M, Meeker AK. Prevalence of the alternative lengthening of telomeres telomere maintenance mechanism in human cancer subtypes. Am J Pathol. 2011; 179(4): 1608-15.
7. Minasi S, Baldi C, Gianno F, Antonelli M, Buccoliero AM, Pietsch T, Massimino M, Buttarelli FR. Alternative lengthening of telomeres in molecular subgroups of paediatric high-grade glioma. Childs Nerv Syst. 2021; 37(3): 809-818.
8. Diplas BH, He X, Brosnan-Cashman JA, Liu H, Chen LH, Wang Z, et al. The genomic landscape of TERT promoter wildtype-IDH wildtype glioblastoma. Nat Commun. 2018; 9(1): 2087.
9. Horbinski C, Miller R, Perry A. Gone FISHing: Clinical Lessons Learned in Brain Tumor Molecular Diagnostics Over the Last Decade. 2011; 21(1): 57-73.
10. Horbinski C, Nikiforova MN, Hagenkord JM, Hamilton RL, Pollack IF. Interplay among BRAF, p16, p53, and MIB1 in pediatric low-grade gliomas. Neuro Oncol. 2012; 14(6): 777-789.
11. Mu Q, Chai R, Pang B, Yang Y, Liu H, Zhao Z, Bao Z, Song D, Zhu Z, Yan M, Jiang B, Mo Z, Loi SC, Tam SS, Chan AK, Wu AR, Liu Z, Poon WS, Ng HK, Chan DTM, Iavarone A, Nam DH, Jiang T, Wang J. Identifying predictors of glioma evolution from longitudinal sequencing. Science Translational Medicine, accepted.
